# Gamma delta T cells in cancer therapy: from tumor recognition to novel treatments

**DOI:** 10.3389/fmed.2024.1480191

**Published:** 2024-12-19

**Authors:** Xinyu Luo, Yufan Lv, Jinsai Yang, Rou Long, Jieya Qiu, Yuqi Deng, Guiyang Tang, Chaohui Zhang, Jiale Li, Jianhong Zuo

**Affiliations:** ^1^The Affiliated Nanhua Hospital, Hengyang Medical School, University of South China, Hengyang, China; ^2^Computer Institute, Hengyang Medical School, University of South China, Hengyang, Hunan, China; ^3^Transformation Research Lab, Hengyang Medical School, University of South China, Hengyang, Hunan, China; ^4^The Third Affiliated Hospital, Hengyang Medical School, University of South China, Hengyang, China

**Keywords:** gamma delta T cell, tumor microenvironment, immunotherapy, adoptive cell therapy, CAR-γδ T cell

## Abstract

Traditional immunotherapies mainly focus on αβ T cell-based strategies, which depend on MHC-mediated antigen recognition. However, this approach poses significant challenges in treating recurrent tumors, as immune escape mechanisms are widespread. γδ T cells, with their ability for MHC-independent antigen presentation, offer a promising alternative that could potentially overcome limitations observed in traditional immunotherapies. These cells play a role in tumor immune surveillance through a unique mechanism of antigen recognition and synergistic interactions with other immune effector cells. In this review, we will discuss the biological properties of the Vδ1 and Vδ2 T subsets of γδ T cells, their immunomodulatory role within the tumor microenvironment, and the most recent clinical advances in γδ T cell-based related immunotherapies, including cell engaging strategies and adoptive cell therapy.

## 1 Introduction

T cells are pivotal in the realm of cancer immunotherapy research (1). They can be classified into αβ T cells and γδ T cells, distinct by their T cell receptor structures. γδ T cells possess a unique TCR composed of γ and δ chains (2), enabling γδ T cells to recognize various antigens, performing multiple roles, including antitumor activities, immune surveillance, and anti-infection capabilities (3–5). Additionally, activated γδ T cells secrete different cytokines depending on the local microenvironment and interact with other cells to participate in the host's antitumor immune response (6, 7). While γδ T cells show immense therapeutic promise, their biological functions and clinical applications remain relatively understudied. Recent research has started to reveal the various roles of γδ T cells in the tumor microenvironment (TME) and explore novel approaches for their clinical application, including the expansion of γδ T cells and the development of chimeric antigen receptor (CAR)-γδ T cells (8–10).

This review provides a comprehensive overview of the current understanding of the mechanisms of γδ T cell recognition and their immunomodulatory role in TME. We will also explore recent advances in γδ T cell-based immunotherapy and discuss the barriers and future directions for γδ T cell research. The primary aim is to connect fundamental research with clinical application to optimize the efficacy of γδ T cell therapy for cancer.

## 2 Tumor recognition mechanisms of γδ T cells

γδ T cells consist of three main functional subsets: Vδ1, Vδ2, and Vδ3 (11, 12). In humans, Vδ2 T cells mainly express the Vδ2 chain and often pair with Vγ9 to form Vγ9Vδ2 T cells, primarily found in peripheral blood (13, 14). Vγ9Vδ2 T cells have been extensively studied due to multiple tumor cell recognition receptors and their ease of expansion *in vitro* (15–17). Vδ1 T cells are the second most abundant type in the peripheral blood (18, 19). These cells recognize MHC class irrelated polymorphic molecules through natural killer group 2 member D (NKG2D) receptors (20). Proteins encoded by MHC class I-chain related genes A and B (MICA and MICB), along with UL16-binding proteins (ULBP) (21), are expressed under conditions of cellular stress, damage, or transformation and bind to NKG2D receptors, serving as “kill me” signals to cytotoxic T cells (22, 23). Vδ3 T cells comprise a relatively rare subpopulation in the peripheral blood, liver, and intestines (24, 25). They multiply in reaction to cytomegalovirus infection and are involved in developing dendritic cells (DCs) and B cells. The diverse distribution of different γδ T cell types in various tissues highlights their versatile function in immune responses. More research has been conducted on Vδ2 and Vδ1 T cells in cancer immunotherapy, so this review specifically focuses on these subsets.

### 2.1 BTN3A1 and BTN2A1 mediate recognition of phosphoantigens by γδ T cells

The process by which γδ T cells identify tumor-associated antigens (TAAs) primarily involves the TCR and NKR pathways (6). Under conditions of cellular stress, Vγ9Vδ2 TCR recognizes phosphoantigens (pAgs) to initiate immune responses (26). pAgs products produced by the isoprene biosynthetic pathway. For instance, a common pAg, isoprene pyrophosphate (IPP), is present in all living organisms. Another potent activator, (E)-4-hydroxy-3-methyl-but-2-enyl pyrophosphate (HMBPP), originating from specific microbes and parasites (27, 28). HMBPP activates the Vγ9Vδ2 T cell receptor much more effectively than IPP (29). The level of pAgs in normal cells is extremely low. However, tumor cell development can lead to the accumulation of endogenous pAgs, making them rapidly identifiable and targetable by Vδ2 T cells. Clinical studies have demonstrated that increasing the IPP levels promotes the activity of farnesyl pyrophosphate synthase in the isoprenoid pathway. Various strategies involve the use of aminobisphosphonates, such as zoledronate (ZOL) and pamidronate, or synthetic pAg analogs to directly activate Vγ9Vδ2 T cells. Studies have shown that γδ T cells exhibit moderate cytotoxicity against tumor cells without pAg. However, when HMBPP or ZOL is added, it induces TCR-dependent cytotoxicity in γδ T cells (30).

**Figure 1:**
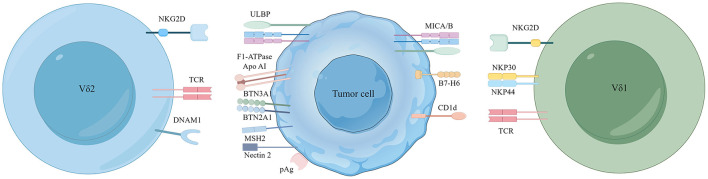
Major receptor-ligand recognition between Vδ2/Vδ1 T cells and tumor cells. Recognition of phosphoantigens by the human Vγ9Vδ2 TCR mainly depends on the synergistic action of BTN3A1/BTN2A1. Vδ2 T cells also recognize a complex of F1-ATPase bound to ApoAI and MutS homolog 2 (MSH2). Nectin-2 from tumor cells can also activate Vδ2 T cells by binding to DNAM-1. The Vδ1 TCR recognizes CD1d and its presented lipids, and its expression of NKp30 and NKp44 receptors can bind to ligands B7-H6. In addition to this, NKG2D receptors expressed on Vδ2 and Vδ1 T cells both recognize MICA/B and ULBP molecular ligands.

Vδ2 T cells do not directly recognize pAgs but depend on the combined action of butyrophilin subfamily 3 member A1 (BTN3A1) and BTN2A1 (31) (Figure 1). The pioneering study by Harly and colleagues first identified a crucial role of BTN3A1 in regulating pAgs responses in Vδ2 T cells (32). BTN3A1 (CD277) and BTN2A1 are members of the butyrophilin family. They are part of the immunoglobulin-like molecules with extracellular segments containing IgV and IgC domains, and intracellular segments consisting of B30.2/SPRY cytoplasmic domains (33). The interaction mechanism among these molecules is still debated, but the prevailing hypothesis supports an “inside-out signaling” model. According to this hypothesis, after the increase of intracellular IPP levels, BTN3A2 or BTN3A3 form heterodimers with a unique surface topology different from the homodimers of BTN3A1. In this process, these heterodimers allow structurally diverse pAg molecules to bind to the intracellular B30.2 domain of BTN3A1, forming a molecular glue complex interface (34). pAgs directly bind to BTN2A1 through this interface. pAgs directly bind to BTN2A1 through this interface. By varying affinities, BTN2A1 articulates onto the Vγ9 chain of the γδ TCR, forming a complex with distinct BTN3A1-V and BTN2A1-V domain topologies (35), initiating TCR-mediated γδ T cell activation (36). This mechanism operates independently of αβ T cells, offering potential for therapeutic development. However, further in-depth studies are required to clarify whether the Vδ2 chain of the Vγ9Vδ2 TCR is involved in recognizing the antigenic process. Recent studies have identified AMPK regulating BTN2A1 and BTN3A interactions within Vδ2 T cells, unveiling a stress-mediated regulatory mechanism that enhances the cytotoxic capabilities of Vδ2 T cells (37). Overall, the mechanism by which Vδ2 T cells recognize TAAs through BTN3A1 and BTN2A1 provides new opportunities for antitumor therapy. The Vg9Vd2 TCR can also recognize F1-ATPase (which binds to apolipoprotein AI, referred to as Apo AI) (38). F1-ATPase is ectopically expressed on the cell membrane of specific tumor cells, for instance human leukemia (K562) cells and lymphoma (Raji) cells. ZOL can bind to ApoA1 as a presenting molecule after high-dose ZOL treatment, enhancing its stimulatory effect on Vδ2 T cells (39). Furthermore, aberrantly expressed MutS homolog two composed complex (MSH2) has also been discovered to mediate γδ T cells recognition, thereby triggering cytolysis of tumor cells (40–42).

### 2.2 Role of NKG2D and its ligands in γδ T cell activation

γδ T cells recognize TAAs not only through the γδ TCR but also through various natural killer receptors (NKR) expression, such as natural killer group 2 member D (NKG2D) and DNAX accessory molecule-1 (DNAM1) (43). NKG2D in Vδ2 T cells binds to MHC class I polypeptide-related sequence A/B (MICA/B) (44), retinoic acid early inducible 1 (Rae-1) and UL16 binding proteins (ULBP) found on tumor cells (45). Concurrently, DNAM1 interacts with Nectin-5, Nectin-2, and the poliovirus receptor (PVR) on the surface of tumor cells. Such interactions mediate the cytotoxic response, targeting killing tumor cells via the perforin-granzyme pathway (46, 47) (Figure 1).

NKG2D, an activating cell surface receptor, is primarily found in cytotoxic immune cells, including NK cells, NKT cells, and specific γδ T cell subsets (43). The ligand for this receptor is absent in normal cells but is frequently present in malignant cells. Upon encountering tumor cells, the Vδ2 T cell subset undergoes rapid proliferation and upregulates NKG2D expression, bolstering immune surveillance (48). Nadia et al. demonstrated that mice deficient in NKG2D have a higher prevalence of highly malignant prostate cancer and promote tumor progression (49). Moreover, a rapid response of NKG2D to its ligand Rae-1 was observed in mouse γδ T cells. Persistent overexpression of Rae-1 downregulates NKG2D expression, thereby attenuating the antitumor functions of T cells (50). Moreover, the DNAM-1 receptor is pivotal in mediating γδ T cell targeting tumor cells. The antitumor response of human γδ T cells strongly correlates with the presence of DNAM-1 ligands on tumor cells (51, 52). The study found that the inhibition of PVR and Nectin-2 led to a marked decrease in the cytotoxic capabilities and cytokine secretion activities of γδ T cells (47).

### 2.3 CD1d is the key driver of vδ1 T cell activation

In human Vδ1 T cells, CD1d has emerged as a critical antigen-presenting molecule (53). CD1d, a glycolipid antigen-presenting molecule, is expressed in various cancers, including renal cell (54), medulloblastoma (55), glioma (56), multiple myeloma (57), breast (58), and prostate (59). The Vδ1 TCR can recognize CD1d and its lipid antigens (5), which may facilitate tumor growth by prompting type 1 NKT cells to release immunosuppressive cytokines, thereby aiding protumor NKT cell subsets (60). Besides CD1d, Vδ1 T cells depend on the expression of NKp30 and NKp44 receptors (61). Researchers have shown that the targeted knockdown of the B7-H6 ligand, bound to the NKp30 receptor, utilizing the CRISPR/Cas9 gene-editing technology significantly diminishes the antitumor response of γδ T cells in acute myeloid leukemia (AML) (62). In addition, it is reported that NKp46 is expressed explicitly on intraepithelial Vδ1 T cells in the intestine (63). Remarkably, the Vδ1 T cells can also recognize MICA/B via NKG2D, and direct binding of MICA/B to Vδ1 has been demonstrated (64). These findings provide new insights into the role of Vδ1 T cells in tumor immunity and offer potential new targets for cancer therapy (Figure 1).

## 3 Immunomodulatory role of γδ T cells in the TME

### 3.1 Cytokine-mediated modulation of γδ T cell functions

**Figure 2:**
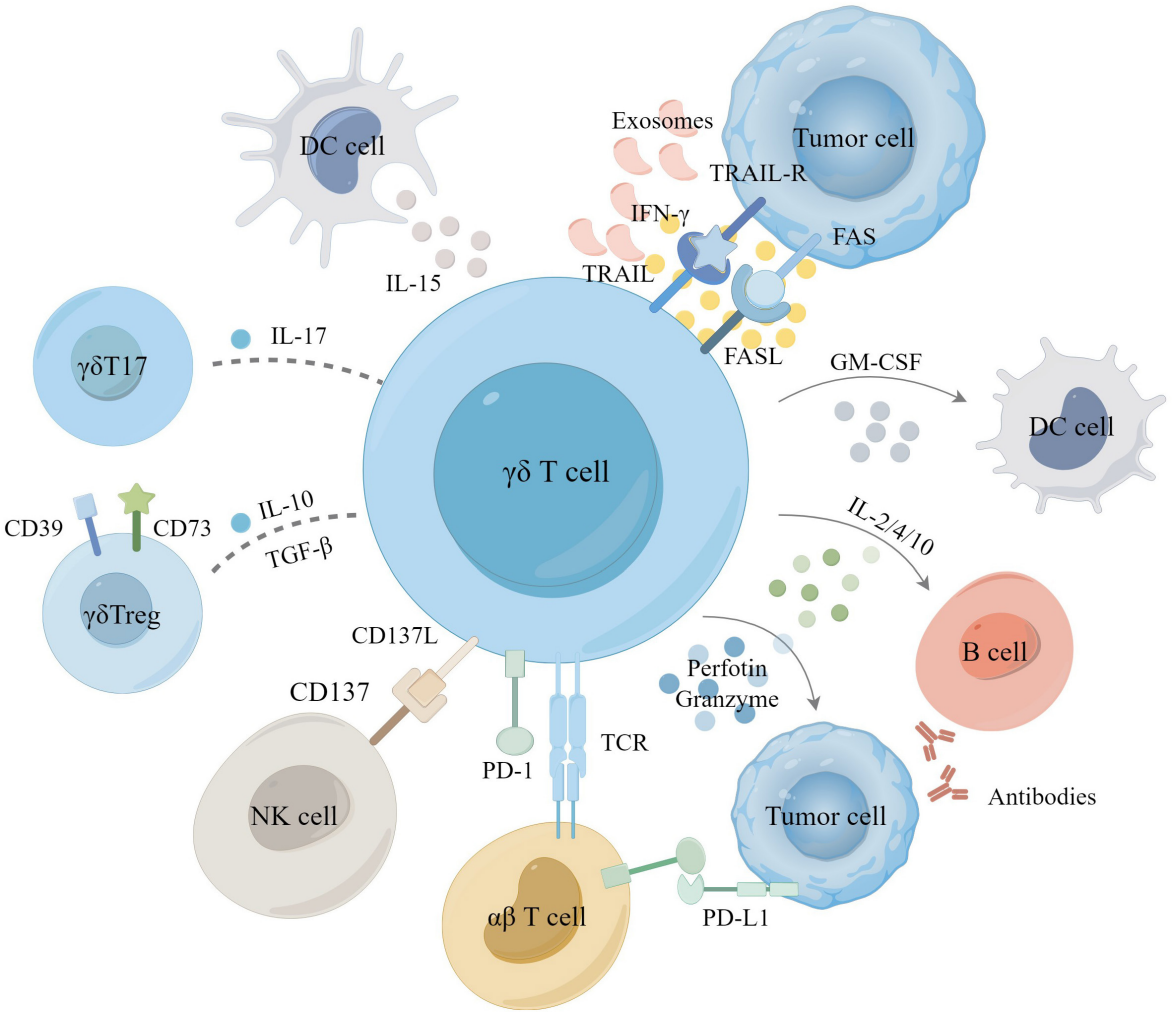
The role of γδ T cell in tumor microenvironment. Activation of γδ T cells induces tumor cell apoptosis primarily through the perforin-granzyme mechanism, Fas/FasL, and TRAIL pathways. Additionally, γδ T cells eliminate tumor cells by releasing cytokines such as IFN-γ and TNF-α, synergizing with the activation of αβ T cells and NK cells and promoting the conversion of antibodies in B cells as well as the antigen-presenting role of DC cells. In certain instances, γδ T cells differentiate into γδ T17 or γδ Treg cells, which secrete IL-17, IL-10, and TGF-β to facilitate the proliferation of tumor cells, playing an immunosuppressive role. Tumor-derived exosomes (TDE), immunosuppressive receptors have also been shown to modulate γδ T cell immunoregulation in TME.

γδ T cells induce apoptosis of tumor cells mainly through the perforin-granzyme mechanism or the Fas/FasL and TRAIL pathways (65, 66). They can also target tumor cells for killing through antibody-dependent cell-mediated cytotoxicity in tumor immunosurveillance (67, 68). γδ T cells stimulate immune responses indirectly by secreting cytokines like interferon-γ (IFN-γ), tumor necrosis factor-α (TNF-α), interleukin (IL)-2, IL-10, IL-12, and IL-15, impacting both tumor cells and the microenvironment (69, 70) (Figure 2).

γδ T cells serve as a primary and early source of pro-inflammatory cytokines upon activation, both *in vitro* and in vivo (71). Activated gd T cells secrete IFN-γ and TNF-α to inhibit tumor cell growth. Upon activation, they secrete IFN-γ and TNF-α, which inhibit tumor cell growth. IFN-γ release stimulates cancer stem cells (CSCs) to upregulate MHC class I molecules and intercellular cell adhesion molecule-1(ICAM-1), enhancing CD8+ T cell-mediated cytotoxicity against tumor cells (72, 73). In the presence of pAgs, IL-15-cultured dendritic cells (DCs) significantly boost the anti-tumor activity of γδ T cells through the secretion of soluble IL-15. This secretion upregulates cytotoxic molecules (CD16) and co-stimulatory molecules (CD80/86) on γδ T cells (74). Adding IL-12 and vitamin C (VitC) to the culture medium significantly enhances proliferation of γδ T cells and production of IFN-γ (75–77). Under the influence of VitC, expanded γδ T cells *in vitro* display heightened antitumor response in preclinical humanized mouse models and tumor cell assays (78).

γδ T cells producing IL-17 and those producing IFN-γ in the TME have opposing effects on patient prognosis (79). Elevated levels of IL-17 are closely linked with tumor metastasis and poor outcomes (80). IL-10 and transforming growth factor-β (TGF-β) within the TME facilitate the differentiation of γδ T cells into different functional subsets, such as γδT17 cells and γδTregs (81, 82). Numerous studies in mice have demonstrated that IL-17 promotes cancer progression through various mechanisms, including promoting angiogenesis, increasing endothelial cell permeability, and upregulating adhesion molecules (83). Liu et al. further reported that in multiple myeloma (MM), bone marrow stromal cells (BMSC) produce CXCL10, recruiting peripheral blood γδ T cells to the bone marrow microenvironment. Hypoxic conditions within the TME promote IL-17 secretion by γδ T cells via the SRC3/RORγt/IL-17 pathway. Interestingly, there are conflicting findings regarding colorectal cancer (CRC) (84). Wu et al. observed high levels of γδT17 cells in γδ T tumor-infiltrating lymphocytes (TILs) in CRC (83). At the same time, Meraviglia et al. found very low IL-17 secretion by γδ T cells in different CRC patient cohorts (85). However, a recent study suggested that IL-17 could be linked to antitumor activity. In a KIT-driven mouse model of gastrointestinal stromal tumor (GIST), γδ T cells were activated and highly expressed programmed cell death protein-1 (PD-1) and secreted IL-17. It was observed that γδT17 cells could be further activated to release IL-17 with tyrosine kinase inhibitors (86). The improved antitumor efficacy indicated that IL-17 might contribute to antitumor effects. Within the TME, γδTregs may suppress γδ T cell proliferation and cytotoxicity by producing immunosuppressive molecules such as IL-10, IL-8, and adenosine (ADO) (87, 88). γδTreg cells exhibit high surface expression of CD39 and CD73 (89, 90), suppressing other effector cells in an ADO-dependent manner. This suppression involves upregulating programmed cell death ligand-1 (PD-L1) and activating the STAT3 signaling pathway in DCs, leading to DCs senescence to promote tumor growth (91).

### 3.2 Innate immunity and antigen presentation of γδ T cells

γδ T cells, recognized for their MHC-independent activity, exhibit innate immune functions and antigen-presenting capabilities, similar to NK cells, DCs, macrophages, and B cells (92, 93). Upon activation, γδ T cells secrete pro-inflammatory cytokines and chemokines, creating an inflammatory environment that promotes the presentation of MHC class I- and II-restricted peptides on tumor cells (94). This enhances the expression of co-stimulatory molecules, such as CD80/86, and robustly stimulates CD4+/CD8+ αβ T cell activation and proliferation (95). Additionally, activated γδ T cells indirectly promote αβ T cell proliferation by co-stimulating NK cells via the ICOS/ICOS-L and CD137/CD137L pathways, thereby increasing IFN-γ and TNF-α production (96). Notably, following IL-21 and HMBPP stimulation, γδ T cells induce B cell homing, migration, and aggregation in lymph nodes, facilitating antibody production and class switching (97–99). Mature DCs synergize with ATP, IPP, and BTN3A1 to activate γδ T cells by secreting cytokines like IL-12, IL-18, IFN-γ, and TNF-α (100, 101). In turn, γδ T cells promote DC maturation by secreting IFN-γ and TNF-α, thereby enhancing the activation of both αβ and γδ T cells and amplifying antitumor responses (102). However, COX-2-expressing MSCs and Prostaglandin E2 (PGE2) from tumor cells can inhibit γδ T cell cytotoxicity (81). Furthermore, galectin-9 on γδ T cells and tumor cells drives the polarization of M2-like tumor-associated macrophages, which secrete immunosuppressive molecules that impede the antitumor activity of γδ T cells (103). In conclusion, understanding the intricate relationship between immunity effector cells and γδ T cells within the TME is crucial for harnessing the therapeutic γδ T cells in cancer treatment.

### 3.3 Exosome-mediated modulation of γδ T cells

The interaction between tumor-derived exosomes (TDEs) and γδ T cell responses within the TME plays a dual role in promoting and inhibiting tumor immunity (104). *In vitro*, stimulation of γδ T cells with TDEs significantly upregulated PD-1 expression, unaffected by miR-21 overexpression or anti-PD-L1 agents, to induce tumor immune escape. Hypoxic TDEs further enhanced the immunosuppressive functions of myeloid-derived suppressor cells (MDSCs) and inhibited γδ T cell proliferation (105). In contrast, gastric cancer cell-derived exosomes enriched with THBS1 enhanced Vγ9Vδ2 T cell cytotoxicity against gastric cancer, increasing the production of IFN-γ, TNF-α, perforin, and granzyme B both *in vivo* and *in vitro* (106). Additionally, exosomes from Vδ2 T cells (Vδ2-T-Exos) activate FasL and TRAIL pathways, effectively killing EBV-associated tumor cells while expanding EBV-specific CD4+ and CD8+ T cells. In a mouse model, administration of Vd2-T-Exos effectively controlled EBV-associated tumors (107). Despite these promising findings, further research is necessary to fully utilize exosomes for enhancing the clinical effectiveness of γδ T cells. A thorough understanding of the exact interactions and optimal utilization of TDEs may lead to more efficacious γδ T cell-based immunotherapies.

### 3.4 PD-1/PD-L1-mediated γδ T cell regulation

While activated γδ T cells can enhance αβ T cell responses, they may also negatively regulate them by upregulating PD-1/PD-L1 (61). Meanwhile, γδT17 cells secrete cytokines like IL-17 and TNF-α, promoting IL-6 secretion and activating the STAT3 pathway, which induces PD-L1 expression and contributes to immunosuppression (108–110). Upon stimulation by ZOL and IL-2, the PD-1 expression of Vδ2 T cells returns to baseline levels after the temporary increase (111). Research shows that PD-1-expressing γδ T cells produce less IFN-γ post-stimulation, reducing their antitumor efficacy (112). In contrast, pembrolizumab treatment rapidly expands γδ T cells, enhancing their recruitment to tumors and IFN-γ and TNF-α secretion (113). Rancan et al. showed that non-Vδ2 T cells are the primary population expressing PD-1, TIGIT, and TIM3 within tumor tissues. Higher transcriptional scores in these cells correlate with improved 5-year survival rates in patients. Additionally, Vδ2– T cells can express 4-1BB, CD39, and CTLA-4, promoting the secretion of IFN-γ, perforin, and granzymes A/K (17). Overall, γδ T cells exert antitumor effects through multiple direct and indirect mechanisms, and their demonstrated function in the tumor microenvironment makes them essential players in cancer therapy.

## 4 Cancer immunotherapy with γδ T cells

γδ T cells are uniquely positioned to recognize and target killing tumor cells, enriched within tumor tissues are correlated with improved clinical outcomes, under-scoring their potential as a promising target for immunotherapeutic strategies. Currently, immunotherapy for γδ T cell tumors primarily involves killing tumor cells by activated γδ T cells using cell engagers. Another approach is adoptive cellular therapies (ACTs), which involves selectively expanding γδ T cells in patients using small molecule pAgs or reintroducing *in vitro*-expanded allogeneic γδ T cells into the human body. Moreover, tumor-targeted activation of CAR-γδ T cells have demonstrated potential in addressing both hematological malignancies and solid tumors (114, 115).

### 4.1 γδ T cell engagers target and kill tumor cells directly

**Figure 3:**
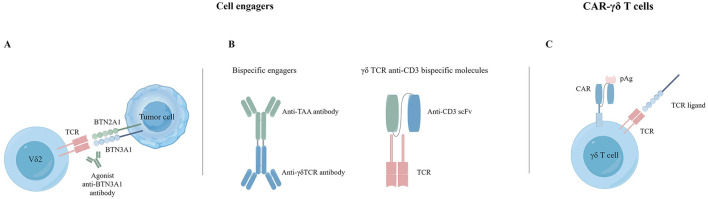
Cancer immunotherapy with γδ T cells. **(A)** Agonist antibodies targeting BTN3A1. **(B)** Bispecific T cell engagers. **(C)** CAR-γδ T cells.

Cell engagers involve using monospecific or bispecific antibodies to connect γδ T cells with tumor targets, resulting in highly targeted tumor destruction. Vγ9Vδ2 T cells can interact with dystrophin via TCR-mediated interactions, and the BTN3A1 antibody induces mimicry of pAg-induced conformational changes to activate the targeting and killing of tumor cells by γδ T cells (116) (Figure 3). Payne et al. demonstrated that anti-BTN3A antibodies induced activation of Vγ9Vδ2 T cells and eliminated inhibition of αβ T cells by BTN3A1 (117). BTN3A1 binds to N-mannosylated residues in CD45 residues on the surface of αβ T cells, hindering their antigen-specific activation. The research on a humanized monoclonal antibody ICT01 targeting BTN3A indicates its ability to rapidly activate non-pAg-dependent Vγ9Vδ2 T cells migrating to tumor tissue. Initial findings from the Phase I/IIa EVICTION trial (NCT04243499; Table 1) of ICT01 in 26 patients with advanced recurrent or refractory cancers revealed a promising safety profile, with no occurrence of serious adverse events. Furthermore, BTN3 antibodies selectively enhance the antitumor function of Vγ9Vδ2 T cells and NK cells without inducing exhaustion of Vγ9Vδ2 T cells caused by ICT01 *in vitro* studies. These findings suggest that treatment with ICT01 can enhance the recruitment and retention of Vδ2 T cells in the TME. Compared to bisphosphonates, ICT01 has a longer plasma half-life, potentially offering greater tumor penetration (118). The EVICTION trial also explores the use of ICT01 in combination with pembrolizumab. Concurrently, an additional clinical trial EVICTION-2 (NCT05307874; Table 1) aims to assess the synergistic effects of ICT01 combined with subcutaneous IL-2 in augmenting T cell responses.

**Table 1:** Current clinical trials of cell engagers.

**Type of therapy**	**Phase**	**Malignancy**	**Status**	**Start**	**Study identifier**
PSMA-V2TCR bispecific antibody	1/2a	R/R mCRPC	Recruting	20220627	NCT05369000/LAVA-1207
CD1d-V2TCR bispecific antibody	1/2a	R/R CLL MM AML	Advanced	20210712	NCT04887259/LAVA-051
BTN3A agonist antibody+VEN/AZA	1/2a	Newly diagnosed AML	Recruting	20200210	NCT04243499
BTN3A agonist antibody+IL-2	1/2a	Solid Tumors	Recruting	20220419	NCT05307874/ICT01-102

Recent work by Mamedov et al. provided further insight into how AMPK regulates the expression of BTN2A1 and BTN3A, thereby influencing the cytotoxicity of γδ and αβ T cells (37). In cellular models and patient tumor tissues, small molecule activation of AMPK increased the expression of BTN2A1-BTN3A complexes and enhanced Vγ9Vδ2 TCR-mediated cytotoxicity. Ongoing clinical trials focused on TEG001 (a hyperactive Vγ9Vδ2 TCR variant, NTR6541) indicate that AMPK agonist treatment heightened the sensitivity of breast cancer organoids and Duodi cells (a typical B lymphoblast) to Vγ9Vδ2 T cell-mediated antitumor response (37). These results emphasize the profound impact of AMPK-dependent metabolic stress-induced upregulation of ligand expression on the interaction of cancer cells with the Vγ9Vδ2 TCR.

Another type γδ T cell engagers consist of two single-chain variable fragments (scFvs) that combine Vδ2 TCR specificity with tumor-targeting VHH antibodies (Figure 3). These engagers target specific molecules such as CD40, CD1d (119), EGFR, and HER2. CD40 is overexpressed on malignant B cells. Researchers have created bispecific T-cell engagers (BiTEs) targeting CD40, such as the CD40-specific Vγ9Vδ2 T cell engager (LAVA-1278). Study have demonstrated its effectiveness in activating and inducing cytotoxicity against malignant B lymphocytes *in vitro* and *in vivo* trials. The fraction targeting the CD40 receptor was observed to inhibit the CD40L-induced pro-survival signaling pathways, subsequently diminishing the resistance of Chronic Lymphocytic Leukemia (CLL) cells toward Bcl-2 inhibitors. A noticeable increase in survival rates was observed in mouse models treated with a combination therapy of both CD40 and Vγ9Vδ2 T-cell dual-specific antibodies, compared to those treated with Vγ9Vδ2 T cells alone (120). CD1d is also expressed upregulated on hematological malignant cells. A BiTE targeting CD1d and Vd2 activates Vg9Vd2 T cells and type 1 NK T cells, leading to the targeting and eliminating malignant and immunosuppressive cells through the perforin/granzyme pathway. In contrast, cytotoxicity against B cells and monocytes is relatively controllable, implying a low risk of on-target-tumor toxicity (60).

Furthermore, the EGFR-Vγ9Vδ2 TCR bispecific engager has successfully activated Vγ9Vδ2 T cells and killed tumor cells *in vitro* and a mouse model, extending the survival of the mice (121). Presently, LAVA-1207, a BiTE that binds with PSMA, activates Vγ9Vδ2 T cells to eliminate PSMA-expressing tumor cells effectively. This agent is currently being evaluated in a Phase I/IIa clinical trial for treating patients with metastatic castration-resistant prostate cancer (NCT05369000; Table 1).

Novel γδ TCR anti-CD3 bispecific molecules (GABs) present an innovative approach to T cell engagers (Figure 3). They link the extracellular domain of the Vγ9Vδ2 TCR, with the CD3 binding domain to form a Vγ9Vδ2 TCR-CD3 complex (122). Traditional BiTEs face challenges in efficacy against solid tumors and often induce relatively high cytokine release syndrome (CRS) toxicity. GABs can potentially reduce toxicity while achieving better efficacy and safety profiles (123). In mouse xenograft models, the Vγ9Vδ2 TCR of GABs redirects αβ T cells to tumor tissues through pAg recognition, significantly inhibiting tumor growth. Cibisatamab (RO6958688) is a GAB that binds carcinoembryonic antigen (CEA) and CD3, promoting T cell infiltration and cytokine release within tumor tissues (124). Notably, cibisatamab can also convert PD-L1-negative tumor cells to PD-L1-positive (125). Moreover, combining cibisatamab with anti-PD-L1 antibodies has demonstrated better control of tumor progression in various tumor types and mouse models (126). Current clinical trials in cell engagers are summarized in Table 1.

### 4.2 Adoptive cellular therapies

#### 4.2.1 Expanding Vγ9Vδ2 T cells for enhanced tumor immunotherapy

Compared to αβ T cells, γδ T cells offer unique advantages in ACT due to their distribution across various tissues, rapid target response, and swift effector function production (127). Vδ2 T cells were the initial subset of γδ T cells tested in ACT studies. ZOL and IL-2 are the most commonly utilized stimulants. ZOL affects the isoprene biosynthesis pathway by explicitly targeting farnesyl pyrophosphate synthase (FPPS), leading to the accumulation of intracellular pAgs (128). This approach effectively expands and activates Vγ9Vδ2 T cells *in vitro*. Another pAg, 2-methyl-3-butenyl-1-pyrophosphate (2M3B1PP), efficiently stimulates and expands Vγ9Vδ2 T cells (129). Several clinical studies have investigated the co-administration of bisphosphonates or synthetic antibodies alongside IL-2, yet the results regarding their effectiveness *in vivo* have generally failed to meet expectations. Yan Xu et al. combined IL-15 and vitC with ZOL and IL-2, significantly expanding Vγ9Vδ2 T cells *in vitro* and improving antigen presentation to effector T cells, thus correlating with intense immune response. In addition, experiments in a mouse transplantation tumor model demonstrated that this regimen effectively suppressed tumor growth *in vivo* and notably extended the survival time of tumor-bearing mice (76). Using bisphosphonate prodrugs and nanotechnology-based Nitrogen-containing bisphosphonate (N-BP) delivery vectors shows great promise in enhancing Vγ9Vδ2 T cell-based immunotherapy. Bisphosphonate prodrugs utilize phosphate groups with chemical masks, facilitating the cellular entry of these compounds (130). On the other hand, nanotechnology-based drugs can enhance the efficacy of killing malignant cells by increasing NKG2D expression in Vγ9Vδ2 T cells and triggering the release of cytokines (131).

#### 4.2.2 Allogeneic Vγ9Vδ2 and vδ1 T cells represent a dual approach to cancer therapy

Allogeneic treatment involves the transfer of allogeneic γδ T cells, which have been grown and activated outside the body, from a healthy donor to a patient with neoplastic conditions. The first team to conduct allogeneic treatment performed a clinical study on 132 patients with advanced liver and lung cancer, utilizing Vγ9Vδ2 T cells from healthy donors. The results demonstrated that after 414 cell infusions, no patients experienced severe adverse effects, with only transient and mild clinical reactions observed in some cases. Furthermore, 18 patients with liver and lung cancer who received multiple cell infusions experienced significantly prolonged survival (76). Vδ1 T cells are also emerging as potential candidates for cancer therapy. However, the lack of reliable methods for their expansion and differentiation has posed a challenge. Sebestyen's team has developed a rapid clinical translation method to produce antitumor effector Vd1 T cells, called Delta One T (DOT) cells. These cells were expanded and differentiated to increase the expression of multiple NKRs, including NKp30, NKG2D, and DNAM-1, and to maintain the expression of immunosuppressive molecules, such as PD-1 and CTLA-4, at low levels or not at all. *In vitro* and xenograft models have demonstrated that DOT cells exhibit significant anti-AML activity (132). The research team has initiated a clinical trial for relapsed/refractory AML (NCT05886491) and is exploring their potential applications in solid tumors. Additionally, a study has suggested that Vδ1 T cells may possess superior tumor cytotoxicity compared to Vδ2 T cells in mouse xenograft tumor models (133). Hence, a deeper understanding of the functional disparities between these two isoforms could aid in fully exploiting their respective clinical benefits. Current clinical trials in ACT using γδ T cells are summarized in Table 2.

**Table 2:** Current clinical trials of expanded γδ T cell subsets.

**Type of therapy**	**Phase**	**Malignancy**	**Status**	**Start**	**Study identifier**
Allogeneic Vγ9Vδ2 T cells	1	R/R AML	Recruting	20200131	NCT03533816
Allogeneic Vδ1 T cells	1/2a	R/R AML	Recruting	20230711	NCT05886491/TAK-012-1501
Allogeneic γδ T cells	1/2	Solid Tumors	Recruting	20210301	NCT04765462
γδ T cell Infusion	1	AML	Recruting	20220321	NCT05015426
Allogeneic expanded γδ T cells with chemotherapy	1	Glioblastoma	Advanced	20200211	NCT04165941
Allogeneic γδ T-lymphocytes	2	R/R AML MDS	Recruting	20220815	NCT05358808/TCB008-001
Expanded γδ T cell infusion	1/2	AML ALL MDS	Recruting	20210912	NCT04764513
Allogeneic γδ T cells combined with targeted therapy and immunotherapy	1	Hepatocellular Carcinoma	No yet	20240426	NCT06364787/NCT06364800
Allogeneic γδ T cells with GD2 chemoimmunotherapy	1	Osteosarcoma Neuroblastoma	Recruting	20231106	NCT05400603
Allogeneic or autologous γδ T cells (DeltEx) combinated with chemotherapy	1b/2	Glioblastoma	Recruting	20230908	NCT05664243
Allogeneic γδ T clls	1	R/R MDS AML	No yet	202501	NCT06463327
ZOL, IL-2 and dinutuximab beta	2	Leiomyosarcoma	Recruting	20211115	NCT05080790

#### 4.2.3 CAR-modified Vδ2 and Vδ1 T cells

Genetically modified γδ T-cells (CAR) are at the forefront of cancer immunotherapy. Initially, CAR-γδ T cell therapies primarily targeted the Vδ2 subset (Figure 3). Around 20 years ago, Rischer and colleagues were the first to describe CAR-γδ T cells. They used recombinant retroviruses to introduce G(D2) or CD19-CARs γδ T cells. These cells were then expanded in a laboratory setting under ZOL activation, resulting in an enriched Vγ9Vδ2 T cell population. Upon encountering antigen-expressing tumor target cells, these cells upregulated CD69 and secreted large amounts of IFN-γ, eliminating Burkitt lymphoma cells *in vitro*. In subsequent studies, Deniger et al. utilized the Sleeping Beauty transposon system for gene transfer to indicate that CD19-CAR-γδ T cells could form a highly polyclonal population with dual specificity. These cells exhibited continuous proliferation, secretion of proinflammatory cytokines, improved lysis of CD19 tumor targets, and demonstrated anti-leukemic activity in xenograft mouse models. Another approach uses mRNA electroporation to modify γδ T cells, which show potent anticancer activity against CD19-positive cancer cell lines *in vitro* and *in vivo* (134).

Compared to Vδ2 T cells, Vδ1 T cells have a reduced sensitivity to their activation-induced cell death (AICD), implying a longer survival time *in vivo* (135). Hence, allogeneic CAR-Vδ1 T cells have recently been developed. DOT cells transduced with CD123-directed CAR showed high efficiency in inhibiting AML growth *in vitro* and *in vivo* (136). A single dose of CAR-DOT cells in combination with IL-15, achieved robust tumor control even after rechallenge. Makkouk et al. has developed CAR-Vδ1 T cells in preclinical studies that were genetically modified to target phosphatidylinositol proteoglycan 3 (glypican-3, GPC-3) and release IL-15 in laboratory conditions. This development aims to treat hepatocellular carcinoma and other solid tumors that may exhibit overexpression of GPC-3. Further research has shown that GPC-3 CAR/sIL-15 Vδ-1 T cells exhibited significant anti-tumor effects in live mouse models without inducing GvHD when used as a standalone treatment (137). Another ADI-001 targeting malignant B cells via the CD20 antigen showed an overall remission rate (ORR) of 75% in eight patients who had received multiple treatments in a Phase I study (NCT04735471; Table 1), with instances of complete remission (CR). ADI-001 exhibited considerable tolerance among subjects without reports of severe adverse reactions, demonstrating a favorable safety profile and substantial preliminary efficacy (138).

While traditional CAR-γδ T cell therapy has shown significant progress in treating certain leukemia and lymphoma patients, its effectiveness in treating solid tumors remains limited (9). Consequently, there remains a persisting necessity to enhance both the structural design and the functional efficacy of CAR-γδ T cells. Recent studies have indicated that second-generation CAR-γδ T cells expressing the CD28 co-stimulatory domain enhance IFN-γ secretion and cytotoxicity against prostate cancer cells. In mouse models, CAR-γδ T cell immunotherapy has been demonstrated to slow tumor growth, and when combined with ZOL, it enhances cytotoxicity and cytokine secretion (139).

Apart from the strategies mentioned above, new therapies based on γδ T cells are continually emerging. Murai et al. have successfully generated nearly unlimited regenerative γδ T cells from γδ T-induced pluripotent stem cells (iPSCs) (140). iPSC-derived γδ T cells (iγδ-T) demonstrate MHC-unrestricted cytotoxicity against cancer cells. However, challenges persist in clinical applications due to using heterologous serum and feeder cells in the iγδ-T induction regimen (141). Lastly, to provide a comprehensive overview of current advances in CAR-γδ T cell immunotherapy, we have compiled ongoing clinical trials as shown in Table 3. These trials cover a wide range of cancer types, further demonstrating the road promise of CAR-γδ T cells immunotherapy.

**Table 3:** Current clinical trials of CAR-γδ T cells.

**Type of therapy**	**Phase**	**Malignancy**	**Status**	**Start**	**Study identifier**
Allogenic CD19-targeting CAR-γδ T cell	1/2a	R/R NHL	Recruting	20221211	NCT05554939
Allogenic B7H3-targeting CAR-γδ T cell	1/2	R/R B7H3 Positive malignant brain glioma	Recruting	20230601	NCT06018363
Allogeneic CD20-specific CAR-Vδ1T cells	1	R/R B-cell NHL DLBCL	Recruting	20210304	NCT04735471/NCT04911478
CAR-γδ T Cells	1/2	R/R Solid Tumors	No yet	20240430	NCT06150885
Allogeneic CD70-specific CAR-Vδ1T cells	1/2	R/R ccRCC	No yet	202309	NCT06480565

## 5 Conclusion

The distinctive antigen recognition mechanism and immunoregulatory function of γδ T cells render them highly advantageous in tumor immunotherapy. Nevertheless, the limited foundational research on γδ T cells restricts their therapeutic efficacy and broader clinical application. Future research should investigate the mechanisms underlying γδ T cell maturation, activation, and proliferation. It is crucial to comprehend the antigen recognition mechanisms of γδ TCRs and to identify TAAs targeted by γδ T cells. Understanding the dynamic functions of different γδ T cell subsets within the complex TME will be pivotal in optimizing their clinical use. Addressing the significant challenges in clinical trials, including the limited expansion of γδ T cells and their exhaustion post-activation, is also essential. Comprehensive development and optimization of γδ T cells from various perspectives are imperative to maximize the therapeutic potential of γδ T cell-based immunotherapy. By overcoming these challenges and leveraging the unique properties of γδ T cells, we can progress toward more effective and personalized cancer immunotherapies.
